# Effect of Wafer Level Underfill on the Microbump Reliability of Ultrathin-Chip Stacking Type 3D-IC Assembly during Thermal Cycling Tests

**DOI:** 10.3390/ma10101220

**Published:** 2017-10-24

**Authors:** Chang-Chun Lee

**Affiliations:** Department of Power Mechanical Engineering, National Tsing Hua University, No. 101, Section 2, Kuang-Fu Road, Hsinchu 30013, Taiwan; cclee@pme.nthu.edu.tw; Tel.: +886-3-516-2410; Fax: +886-3-572-2840

**Keywords:** 3D integrated circuits, microbump reliability, wafer level underfill, temperature cycling test, finite element analysis

## Abstract

The microbump (μ-bump) reliability of 3D integrated circuit (3D-IC) packaging must be enhanced, in consideration of the multi-chip assembly, during temperature cycling tests (TCT). This research proposes vehicle fabrications, experimental implements, and a nonlinear finite element analysis to systematically investigate the assembled packaging architecture that stacks four thin chips through the wafer level underfill (WLUF) process. The assembly of μ-bump interconnects by daisy chain design shows good quality. Results of both TCT data and the simulation indicate that μ-bumps with residual SnAg solders can reach more than 1200 fatigue life cycles. Moreover, several important design factors in the present 3D-IC package influence μ-bump reliability. Analytical results show that the μ-bump’s thermo-mechanical reliability can be improved by setting proper chip thickness, along with a WLUF that has a low elastic modulus and a small coefficient of thermal expansion.

## 1. Introduction

Given that chip-stacking packages, combined with either silicon-based or glass/ceramic-based interposers, are promising frameworks [1,2,3,4,5] for three-dimensional integrated circuit (3D-IC) integrations [6,7,8], microbump (μ-bump) reliability must be enhanced. Processes, such as chip grinding, position adjustment and planarity of assembly, the formation of through-silicon via (TSV), and the composition and dimensions of μ-bumps, should be emphasized to improve the functionality of 3D-IC packages. Consequently, the prevention of fracture failure in μ-bumps during fabrication and the maintenance of their long-term reliability is crucial. At present, μ-bump arrays are placed into an underfill after assembly through the capillary action of traditional flip-chip (FC) technology. In this process, however, voids may surround the μ-bumps in the underfill. Moreover, the bump height or gap between the Si substrate and the stacked chips narrows progressively, thereby deteriorating the μ-bump reliability. This issue can be addressed completely by wafer level underfill (WLUF), using the thermal compression approach [9,10,11,12,13,14,15,16]. The main procedures of the WLUF process are first, to attach WLUF film on a silicon wafer. Subsequently, another chip with μ-bumps is aligned and assembled to the above-mentioned diced silicon wafer, by using a thermal compression bonding approach. Several studies have reported on the applications and characteristics related to underfill and μ-bump reliability [17,18,19]. Shen et al. performed numerical analyses to investigate the mechanics of microbump failure in 3D microelectronic packages under a shear load; the loading mode with a superimposed tension or compression on shear easily influenced the ductility of the concerned solder [17]. By using finite element analysis (FEA), a reliability study was demonstrated by Che et al., involving the architecture of silicon-less interconnection technology (SLIT), in which the results indicated that advanced packaging with SLIT technology shows higher solder joint fatigue life than packaging with through-silicon interposer technology, under thermal cycling conditions [18]. Moreover, the significant influence of the properties of underfill on the thermal mechanical reliability of 3D-IC packaging was emphasized [19]. However, μ-bump reliability has rarely been analyzed for the chip stacking of 3D-IC packages using WLUF in temperature cycling tests (TCT). Thus, the current study fabricates a vehicle to validate the capability and related reliability of the chip-on-chip packaging structure with four thin chips under a TCT load ranging from −55 °C to 125 °C. Moreover, μ-bump reliability is estimated through a nonlinear, thermo-mechanical simulation based on FEA. The mean failure cycles of the μ-bump can be predicted accurately, based on the Coffin-Manson empirical relation and through comparison with testing data. Furthermore, the current study systemically estimates the effects of several important design factors, including stacked chips, the elastic modulus (*E*), and the coefficient of thermal expansion (CTE) of WLUF, on the fatigue lifespan of μ-bumps through reliable simulation methodology.

## 2. Temperature Cycling Test of 3D-IC Packaging

### 2.1. Assembly of Stacked Chips with WLUF

**Figure 1:**
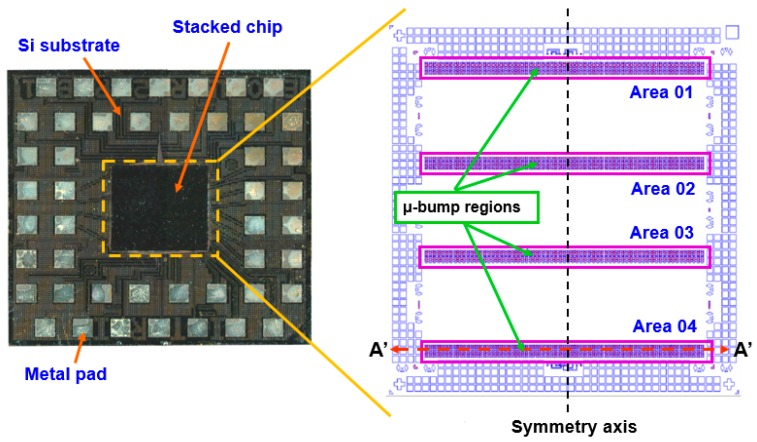
Small stacked chips mounted at the center of a large Si substrate using FC technology and the water level underfill (WLUF) process.

**Figure 2:**
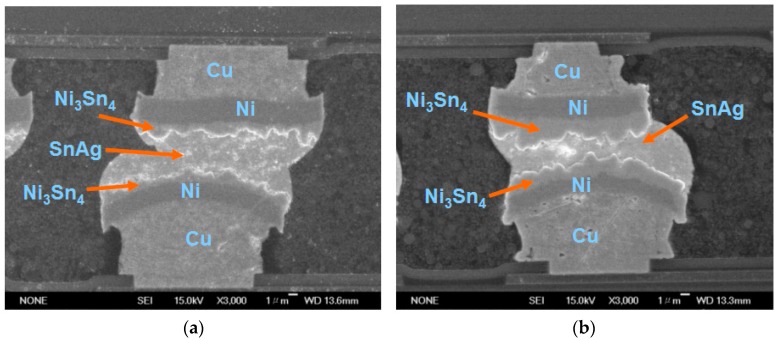
SEM images of the bottom μ-bump array in a four-chip stacking structure generated through four reiterations of the WLUF assembly processes [12]: (**a**) After the bonding of the first chip; (**b**) after the bonding of the fourth chip.

To estimate the reliability and precision of μ-bumps using the proposed simulation methodology, a testing vehicle was fabricated and a related TCT experiment was conducted. Figure 1 shows the top view and the layout design of the arrangement of the chip-on-chip packaging specimen, which is composed of four 50-μm-thick stacked chips. Notably, the TSV in the specimens is not configured to focus on the stability of the μ-bump assembly during the application of the WLUF thermo-compression approach. The detailed assembly process conditions are described as follows. First, a 20 μm laminated WLUF was coated on a 50 μm wafer with array-type μ-bumps. It is notable that the WLUF was composed of a 50% Silica filler, epoxy material, and flux agent. Second, the foregoing wafer was diced into numbers of chips. These chips, with the designs of trace lines, are fabricated and provided by Industrial Technology Research Institute (ITRI) in Taiwan. Third, a thermal compressing bonding was performed by a FC bonder with a pressure of 0.753 MPa and an elevated temperature of 260 °C. The total duration was about 1 min, composed of 5 s and 10 s, for melting solder and pre-heated stages, respectively. Finally, a post curing procedure for WLUF was implemented to complete the whole assembly process. The four stacked chips (each 5.1 mm × 5.1 mm) were mounted on top of the Si substrate (with an area of 16 mm × 16 mm). As indicated in Figure 1, the surface of the stacked chip is divided into four regions, with more than 3000 array-orientation μ-bumps. A fine pitch of 30 μm was applied to the μ-bumps to determine the manufacturing capability for high-density assembly. Moreover, array-type electrical pads, which are part of the daisy-chain design, were installed at the peripheral margin of the Si substrate, to assess electrical performance when the TCT loads were applied. To meet the developed mainstream of 3D-IC packages, a small form factor was necessary. Hence, this study considers the narrow gap in chip assembly, which is mainly determined by μ-bump height. The use of traditional underfill to completely encapsulate the underfill through capillary action is complicated. By contrast, a pressure of 0.753 MPa combined with an elevated temperature of 260 °C had to be applied and maintained throughout the proposed WLUF process. Following assembly, μ-bumps were individually generated from the chip and Si substrate sides. They consisted of a 6-μm-thick Cu stud, a 3.5-μm-thick nickel layer, and an approximately 1.0-μm-thick Ni_3_Sn_4_ intermetallic compound (IMC). In addition, the intermediate zone of the μ-bump contained a 2.5-μm-thick lead-free solder remnant. An enlarged view of the μ-bump assembly is displayed in the scanning electron microscopy (SEM) images in Figure 2 after the A′-A′ dash line presented in Figure 1 was cut. After the fourth chip was bonded, the thickness of Ni_3_Sn_4_ increased (Figure 2b) as a result.

### 2.2. Reliability Experiment Results

**Figure 3:**
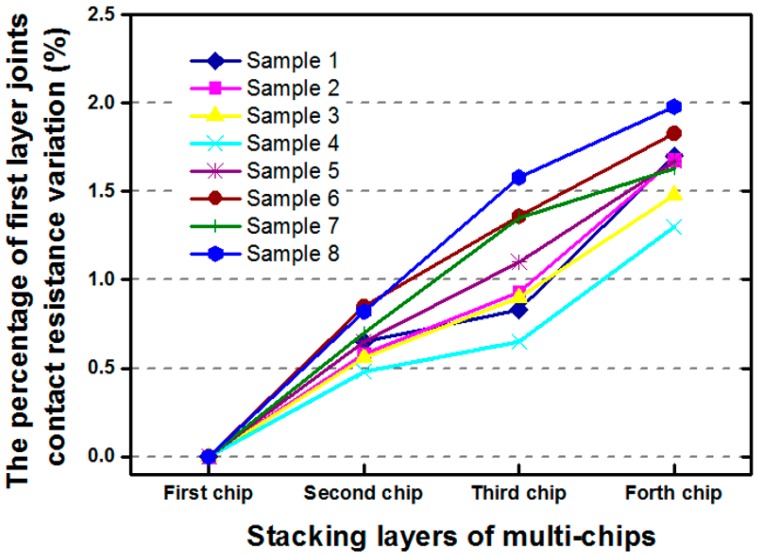
Variation in the contact resistance of the daisy chain at the bottom μ-joint array during multi-chip stacking processes.

**Figure 4:**
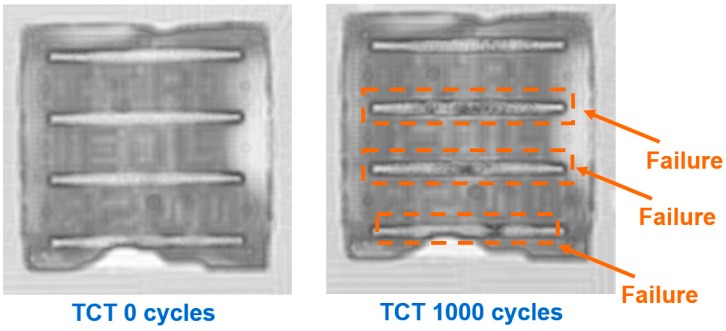
Comparison of satellite acoustic technology (SAT) images obtained at the beginning of the experiment (zero cycle) and those captured after 1000 temperature cycling test (TCT cycles).

To stabilize the stacking of four chips, the daisy-chain design was implemented on the bottom layer of the μ-bump array. Thus, the electrical characteristics could be determined. Figure 3 depicts the variation in contact resistance for eight specimens after each layer of 50-μm-thick chips was stacked. The contact resistance of the μ-bump within the array at the left-upper corner of the first chip was measured by using a 4-point kelvin probe. Typical values of the measured contact resistance were between 40~60 mΩ. In this research, a measured range from 45.5 mΩ to 54.5 mΩ was acquired after assembling the four stacked chips. The measured reproducibility was extremely fine when the first chip was mounted. Moreover, contact resistance varied by only ~2% in the proposed packaging vehicles. In the reliability test, under temperature cycles between −55 °C and 125 °C, the mean number of failure cycles for the first chip on the Si-substrate was ~1250, given 42 specimens of WLUF assembly technology. To determine the primary failure mechanism and the potential collapse locations, the scanning acoustic tomography (SAT) images at the beginning of the reliability test were compared with those obtained after a maximum of 1000 sustained TCT cycles, as shown in Figure 4. After testing, the four regions of the μ-bump array were patterned with many dark spots, thus indicating that μ-joints sustained fractured breaks. The Coffin-Manson relationship had to be incorporated into a simulation methodology to address this occurrence and to estimate the fatigue life of μ-bumps. This technique is detailed in the following section.

## 3. Failure Analysis and the Estimation of Fatigue Lifetime in μ-Bumps

### 3.1. Coffin-Manson Relationship in Lead-Free Bumps

μ-bumps are composed of lead-free solders. After TCT, the IMC does not become entirely brittle, but rather retains some of this solder. Therefore, the Coffin-Manson relationship, based on the strain energy method is applied, to predict the μ-bump reliability of the present 3D-IC package, containing four stacked chips [20,21]. The proposed simulation approach is integrated with a low-cycle fatigue analysis and is described as follows. First, the empirical equation is expressed as:

(1) $$\Delta \varepsilon_{\gamma }N_{f}^{\lambda }=D$$

Moreover, the conversion of the plastic shear strain extent, $\Delta \varepsilon_{\gamma }$, from an entire thermal cycle to the incremental, averaged equivalent plastic strain, $\Delta \varepsilon_{\mathrm{ep}}$, is given by

(2) $$\Delta \varepsilon_{\gamma }=\sqrt{3}\;\Delta \varepsilon_{\mathrm{ep}}$$

where substance coefficients for the fatigue ductility coefficient (D) and fatigue ductility exponent (*λ*) were obtained using the least-squares method. Given the 96.5Sn3.5Ag lead solder, the constants of D and *λ* were 21.9 and 0.93, respectively, as per Equation (1) [22]. By rewriting Equation (1), according to the transformed relation of Equation (2), the equation that describes the connection between $\Delta \varepsilon_{\mathrm{ep}}$ and the fatigue life cycles (N_f_) for the lead-free solder could be restated further, as follows:

(3) $$N_{f}=15.306\;{\left(\Delta \varepsilon_{\mathrm{ep}}\right)}^{-1.075}$$

Once $\Delta \varepsilon_{\mathrm{ep}}$ converged during a temperature cycle via the FEA, the fatigue life (N_f_) of the μ-bump was predicted. The incremental equivalent plastic strain was adopted for the Coffin-Manson relationship because the shrunken bumps constructed using FC technology [23] contribute to normal stress during TCT.

### 3.2. Finite Modeling Assembly of Stacked Chips Using WLUF

**Figure 5:**
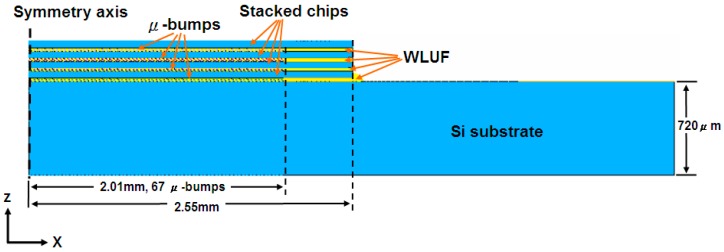
Half symmetry of a finite element model for a stacked chip three-dimensional-integrated circuit (3D-IC) package assembled using four chips.

**Figure 6:**
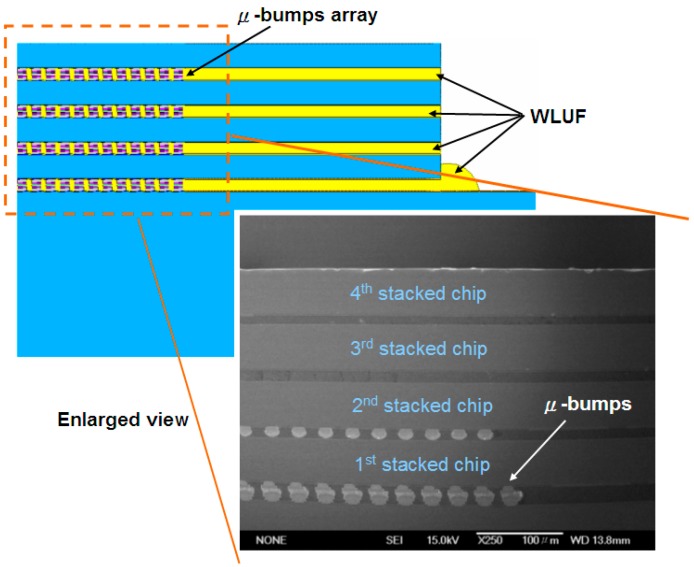
Enlarged cross-sectional views of specimens in the critical bump regions. The peripheral area is rich in WLUF.

**Figure 7:**
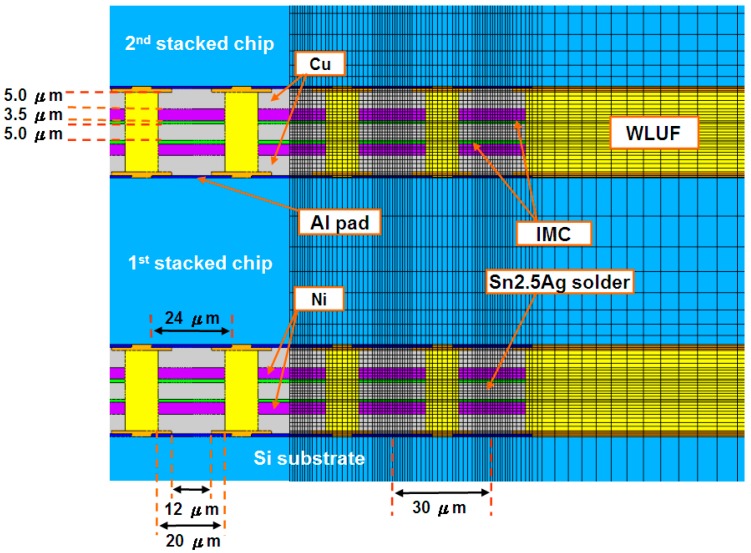
Detailed dimensions, components, and finite element mesh of the μ-bump framework used in the temperature cycling simulation of four-chip stacking, which was assembled through the WLUF thermo-compression procedure; IMC: intermetallic compound.

This research considers two types of chip-stacking 3D-IC packaging structures in the construction of a finite element model. The first type validated failure mode and estimated μ-bump reliability by mounting an initial layer of 50-μm-thick stacked chips. The second type predicted the fatigue lifetime of the μ-bump in the first two layers of the proposed package architecture with four stacked chips. As the second to the fourth stacked chips were bonded, identical WLUF procedures were applied sequentially. As a result, the layout of the entire structure was bi-axially symmetrical. The complexity of these components can generate critical problems in finite element modeling and subsequent computer time. Thus, a 2D simulated model was constructed along the cross-section by cutting the A′-A′ line (Figure 1) that was far from the packaging center. Given the geometry symmetry shown in Figure 5, only half of the model was necessary. In chip assembly, the Si substrate used measured 720 μm. Each stacked chip had an area of 2.55 mm × 2.55 mm and was 50 μm thick. The length of the μ-bump array was 2.01 mm, and this array consisted of 67 μ-bumps with a fine pitch of 30 μm. Hence, WLUF was rich at the peripheral margin of the gap between the first chip and the Si substrate, as well as among the stacked chips. Figure 6 provides an enlarged view of the μ-bumps after chip stacking in both FEA [24,25] and the actual specimen. Figure 7 exhibits the detailed dimensions, components, and finite element mesh of the μ-bump framework used in the temperature cycling simulation. When chips were stacked in four reiterations of the WLUF process, the Ni_3_Sn_4_ IMC displayed a thickness of ~1 μm between the 5 μm thick lead-free solder and the 3.5 μm thick nickel layer. Table 1 lists the material properties of the proposed 3D-IC package used in the nonlinear FEA. Furthermore, a bi-linear model of stress/strain relation was adopted for the Cu stud. The fatigue behavior of μ-bumps was also investigated, based on the temperature dependence of the multi-linear mechanical model for a lead-free solder. The mechanical constraints of the boundary conditions of FEA (Figure 5) are explained as follows. The *x*-directional displacements of the nodes along the symmetry axis of the model were fixed. For the node at the bottom of this axis, all degrees of freedoms were set to prevent rigid body motion during the analysis. On the assumption that intrinsic stresses, resulting from packaging assembly, are totally eliminated, the analytical structure was considered stress-free at room temperature, and the thermal cycling load was subject to a temperature range of −55 °C to 125 °C. At least three cycles were performed to numerically converge the incremental averaged equivalent plastic strains in this FEA investigation.

### 3.3. Failure Mode and Comparison of the Simulated Predictions and the Experimental Results in Terms of the Fatigue Lifetimes of 3D-IC Packaging

**Figure 8:**
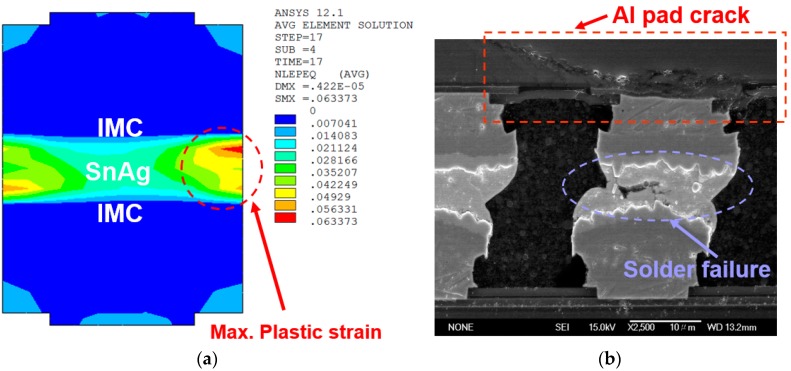
Comparison of the FEA and the experimental examination of four-chip stacked packaging in terms of failure mode: (**a**) Contour of the maximum equivalent plastic strain in the critical μ-joint; (**b**) Solder failure of the critical μ-joint after TCT [12].

The experimental result of TCT indicated that the assembly of a one stacked chip takes 1250 cycles, at approximately 50% of the accumulated failure rate. In eight four-chip stacking structure specimens, more than 1200 cycles were obtained for the μ-bump fatigue lifespan using WLUF. The cross-sections of μ-bumps were examined, as shown in Figure 8b, to determine the failure mechanisms of the proposed vehicles. Potential failure causes were either Al pad fracture or cracking of the μ-bump near the bonded interface between the IMC layer and the lead-free solder. The former fracture mechanism occurs in the wide region of the μ-bump array, as indicated in the SAT check in Figure 4. Enhancing the adhesion of the Al pad and of the neighboring bonded thin films resolves the failure mode of the Al crack by improving the fabrication processes. However, the latter failure mode depends on the long-term reliability performance of μ-bumps in the present packaging structure. Therefore, this research estimated the failure mode and the lifespan of μ-bump fatigue through nonlinear FEA. Figure 8a depicts the contour of the equivalent plastic strain at the outermost edge of the critical μ-bump. Moreover, the analytic results indicated that the maximum principle stress of 40.7 MPa for Ni_3_Sn_4_ IMC occurred at an elevated temperature (125 °C) during the loadings of TCT. As compared with the thickness-dependent critical strength of Ni_3_Sn_4_ IMC (65~85 MPa) [26], the IMC failure mode was difficult to obtain until the foregoing IMC thickness increased to ~4 μm. In this, meanwhile, the critical strength for Ni_3_Sn_4_ IMC was lower than 40 MPa. Furthermore, the predicted location of the maximum value of equivalent plastic strain in the critical μ-bump was identical to the experimental result. The number of mean cycles to failure in the assembly of four 50-μm-thick chips was predicted to be 1324 when a stable 1.57% incremental equivalent strain was substituted into Equation (3), which agrees with the experimental data from TCT. This strain was obtained through a simulated analysis. Therefore, the proposed simulation methodology is highly reliable. Accordingly, the following section analyzes and discusses the effects of several geometric and material characteristics of WLUF on the μ-bump reliability of the 3D-IC package with four stacked chips.

## 4. Geometric and Material Influences of 3D-IC Packages on μ-Bump Reliability

### 4.1. Warpage Induced during Temperature Cycling Loads

**Figure 9:**
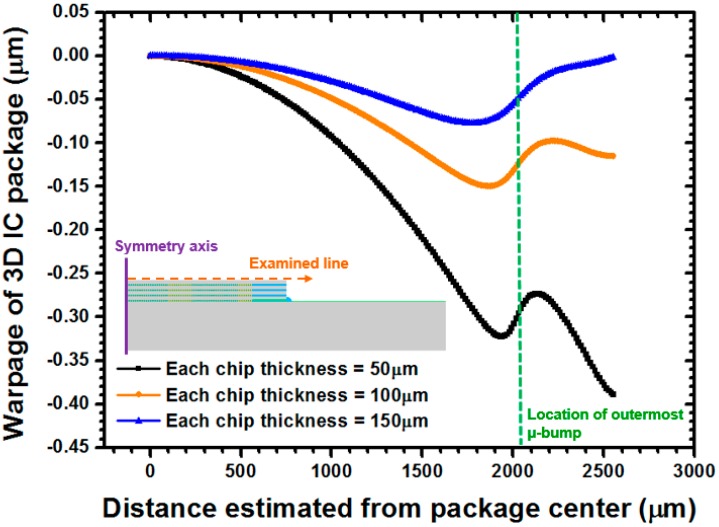
Estimated warpage of the 3D-IC package under WLUF, after the implementation of a stable temperature cycling load, given different stacked chip thicknesses (examined at 25 °C).

In conventional FC technology, the thermal strain on solder joints because of the CTE mismatch between silicon chips and printed circuit boards can be reduced significantly by filling the underfills among them. Shearing stress usually dominates the occurrences of fractures in FC bumps. Unlike the aforementioned situation, however, the stress/strain distribution and structural deformation of the framework of the present package with chip stacking can be determined, based on the complicated material characteristics of the components (i.e., μ-bump array and WLUP) given the identical characteristics used in the chips and in the assembled substrate. Thus, the warpage of an entire package under temperature cycling load must be investigated to further interpret the mechanical behavior of a critical μ-bump. Figure 9 shows the variation in warpage from the interiors to the edges of several stacked chips as judged against the baseline at the central point. A convex-type warpage was observed in all concerned chips in the μ-bump array, at all thicknesses, because of a significant bending effect that was induced by major shrinkage in the μ-bump composition along the horizontal direction. In a highly flexible 50 μm thick chip, the maximum magnitude of approximately −0.322 μm was near the outermost μ-bump. This effect of this warpage lessened with thicker and more rigid chips; maximum warpage decreased sharply from −0.322 μm to −0.077 μm, as the chip thickness increased from 50 μm to 150 μm. Furthermore, the warpage at the WLUF-rich region intensified when the chip was thinner than 50 μm. This issue can be resolved by arranging the μ-bump array such that additional dummy bumps are situated at the peripheral region of stacked chips without μ-bumps, to preserve co-planarity during the subsequent chip stacking process.

### 4.2. Effect of Stacked Chip Thickness

**Figure 10:**
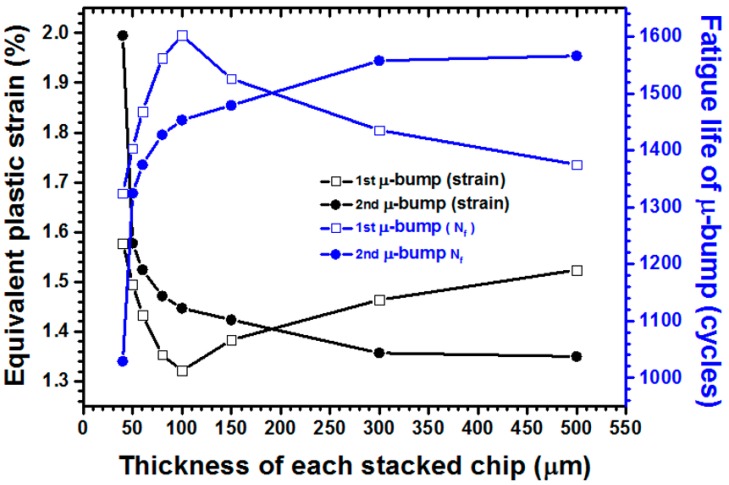
Curves of maximum equivalent plastic strain and predicted fatigue cycles for the outermost μ-bumps in the first and second layers of stacked chips versus various chip thicknesses.

The effects of stacked chips of different thicknesses (i.e., 40, 50, 60, 80, 100, 150, 300, and 500 μm) on bump reliability were parametrically determined during the assembly of four chips. Figure 10 presents the simulated results, although it displays only the curves of the first two μ-bump layers to enhance mass reproducibility and to simplify perusal. The equivalent μ-bump plastic strain increased particularly when the chip was thinner than 100 μm, given that a more flexible thin chip can easily induce severe thermal deformation in the package structure. In the first layer of the outermost μ-bump, plastic strain increased significantly, from 1.32% to 1.58%, when thickness decreased from 100 μm to 40 μm. Thus, fatigue lifetime was expected to decrease from 1602 cycles to 1325 cycles. The μ-bump reliability of the second bump layer, which was enveloped by WLUF, was predicted to deteriorate further. Given a similar range of chip thicknesses, the concerned lifespan decreased from 1453 cycles (1.45%) to 1029 cycles (1.99%). This scenario can be attributed to the fact that μ-bumps arranged in a high stacked chip layer sustain heavy deformation because of the flexibility of the primary stacked chip. Chips thicker than 100 μm are rigid and are expected to transform the dominant type of driving force to a shear mode, applied to the μ-bumps during TCT. Furthermore, the shearing force caused by the CTE mismatch between the silicon chip and the WLUF-rich μ-bump array dominates the failure mode and package reliability. As shown in Figure 10, the fatigue life cycles of the μ-bumps in the bottom layer of stacked chips decreased from 1602 cycles (1.32%) to 1374 cycles (1.58%) when thickness increased continuously from 100 μm to 500 μm. By contrast, strain was estimated to decrease in the second layer of the outermost μ-bump because the rigidity of the chips assembled at the top surface was slightly higher than that of the chips in the first μ-bump layer. Consequently, the calculated μ-bump reliability increased to 1566 cycles (1.35%), based on the Coffin-Manson relationship.

### 4.3. Effect of WLUF Elastic Modulus

**Figure 11:**
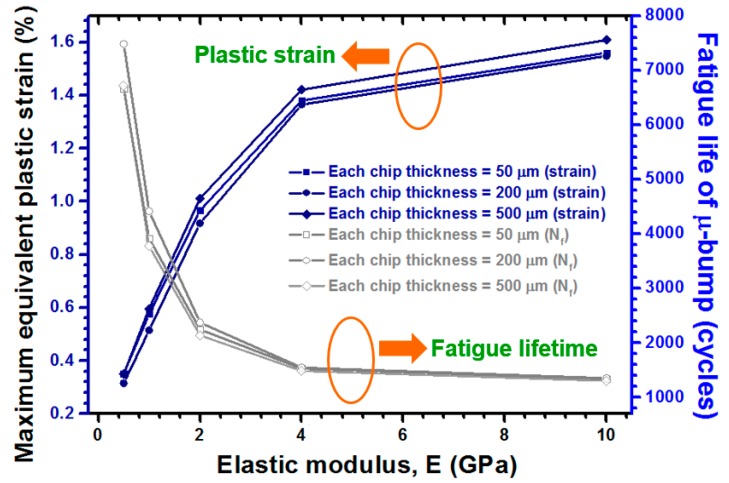
E of WLUF for maximum equivalent plastic strain and the corresponding estimated fatigue lifetime of the critical μ-bump in the first layer of stacked chips.

The E effect of WLUF was systematically examined, over a range of 0.5 GPa to 10 GPa, against stacked chips with several thicknesses. As simulated in Figure 11, the maximum equivalent plastic strain of the μ-bumps dropped significantly from 1.56% (1340 cycles) to 0.35% (6655 cycles) with 50 μm thick chips. As with the traditional underfills used in FC technology, the concentration of stress/strain on the μ-bumps can be distributed effectively with WLUF. This protective effect was pronounced when the E of WLUF was low. μ-bump reliability decreased rapidly as the E of WLUF exceeded 4 GPa. The important difference of the foregoing statement, compared with the preferred high modulus of underfills for chip-on-package, is mainly due to the consideration of the entire thicknesses, composed of stacked chips and filled WLUFs. As the E of WLUF became higher, the equivalent rigidity for the μ-bump arranged at the bottom layer of the silicon chip was larger. Accordingly, a significant shearing deformation mode appeared, due to a serious CTE mismatch among WLUF adjacent to the μ-bumps and assembled upper silicon chips. Thus, the influence of chip thickness, as indicated in this figure, agrees with that obtained through the analysis presented in the previous section.

4.4. μ-Bump Reliability Determined by the CTE of WLUF

**Figure 12:**
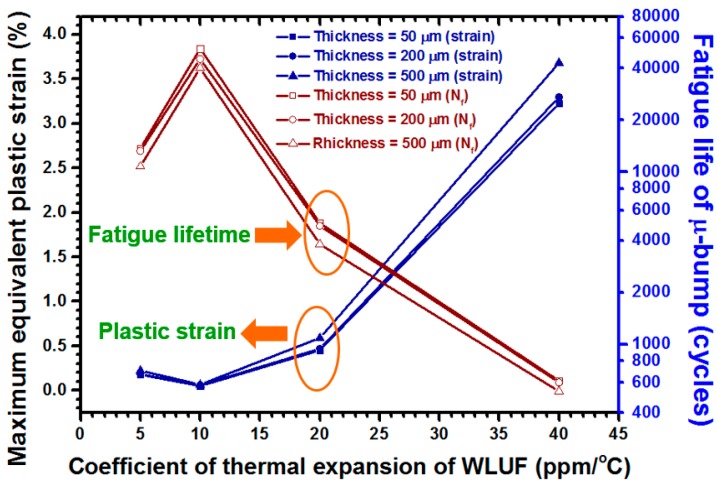
Maximum equivalent plastic strain and fatigue lifetime estimation for the outermost μ-bump in the first layer of stacked chips, in the present 3D-IC package, given several CTEs of WLUF.

The magnitude of thermal stress/strain induced in the package structure by TCT loading must be managed, because it typically dominates the failure causes and long-term reliability of μ-bumps. As an important design factor of WLUF, the effect of CTE on the present 3D-IC package with four-chip stacking was therefore analyzed. In this study, CTEs at 5, 10, 20, and 40 ppm/°C, were examined with the WLUF elastic modulus fixed at 5.6 GPa. Figure 12 displays the simulated results of both the maximum equivalent plastic strain and its corresponding fatigue lifespan, in relation to the critical μ-bump. When CTE was at 10 ppm/°C, strain was minimal at 0.051% and more than 50,000 cycles were observed at inspected WLUF points. Hence, the induced deformation of the whole packaging structure should have been relatively small and the mechanical strain of μ-bump at this moment was presumed to be relatively low. However, μ-bump strain increased sharply to a maximum of 3.68% (533 cycles) when the CTE of WLUF reached 40 ppm/°C, thereby resulting in significant CTE mismatches among the components of the present 3D-IC package. Therefore, a decrease in the CTE of WLUF can enhance μ-bump reliability.

## 5. Conclusions

In this study, four 50-μm-thick chips were stacked using the proposed WLUF process. To determine the quality of the μ-bump connection, as well as the subsequent tolerance of warpage, induced during the WLUF thermal compression procedure, this study implemented an electrical check via the daisy chain method. The improvement in resistance was slight but acceptable. In addition, the Coffin-Manson relationship, associated with lead-free solders, was combined with non-linear FEA to estimate the μ-bump reliability. As per the validation of TCT experimental data, under temperatures ranging from −55 °C and 125 °C, the proposed simulation methodology was highly reliable. The four 50-μm-thick chip packages exceeded 1200 cycles. The FEA-predicted occurrence of fracture at the region near the IMC/lead-free solder interface of the critical μ-bump was consistent with that obtained in actual testing. Based on the comparisons, several geometric and material property effects, including stacked chip thickness, *E*, and the CTE of WLUF were determined. The results show that flexibility can be enhanced by scaling chip thickness down to 100 μm. This high flexibility can, in turn, deteriorate the μ-bump reliability. Hence, WLUF should be characterized by a low E and a small CTE, based on the analysis results of this study. Furthermore, it is believed that the thermal compression approach of WLUF could overcome the filled void issue, while the pitch of microbumps is continually scaled down to the range of 10~20 μm.

## Figures and Tables

**Table 1:** List of material properties used to estimate the presented nonlinear finite element analysis (FEA).

Materials	Young’s Modulus (E)	Coefficient of Thermal Expansion (CTE) (ppm/°C)	Poisson’s Ratio
Si	169.5 GPa	3	0.28
Cu	E = 122 GPa	17	0.35
	Reference temperature (T) = 25 °C		
	Yield stress = 173 MPa		
	Tensile strength = 1.2 GPa		
Passivation (Si_3_N_4_)	155 GPa	5	0.28
Wafer-level underfill	5.6 GPa@25C	29.2	0.33
SnAg solder	Temperature dependence	22.5	0.4
IMC (Ni_3_Sn_4_)	85.6 GPa	−40 °C:16.3	0.31
		25 °C:17.6	
		50 °C:18.1	
		125 °C:19.3	
Ni	186	12.5	0.342
Al	72 GPa	24	0.36
